# Artificial intelligence vs COVID-19: limitations, constraints and pitfalls

**DOI:** 10.1007/s00146-020-00978-0

**Published:** 2020-04-28

**Authors:** Wim Naudé

**Affiliations:** Technology, Innovation, Entrepreneurship and Marketing, RWTH Aachen University, Kackertstrasse 7, 52072 Aachen, Germany

**Keywords:** COVID-19, Data science, AI, Surveillance, Public health

## Abstract

This paper provides an early evaluation of Artificial Intelligence (AI) against COVID-19. The main areas where AI can contribute to the fight against COVID-19 are discussed. It is concluded that AI has not yet been impactful against COVID-19. Its use is hampered by a lack of data, and by too much data. Overcoming these constraints will require a careful balance between data privacy and public health, and rigorous human-AI interaction. It is unlikely that these will be addressed in time to be of much help during the present pandemic. In the meantime, extensive gathering of diagnostic data on who is infectious will be essential to save lives, train AI, and limit economic damages.

## Introduction

The COVID-19 pandemic poses a number of challenges to the Artificial Intelligence (AI) Community. Among these challenges are “Can AI help track and predict the spread of the infection?”, “Can AI help in making diagnoses and prognoses?”, “Can it be used in the search for treatments and a vaccine?” and “Can it be used for social control?” This paper is an attempt to provide an early review of how AI have so far been contributing in this regard, and to note limitations, constraints, and pitfalls. These include a lack of data, too much (noisy and outlier) data, and growing tension between data privacy concerns and public health imperatives.

To start out, let me discuss the actual and potential uses of AI in the fight against COVID-19.

## Tracking and prediction

AI can in principle be used to track and to predict how the COVID-19 disease will spread over time and space. In fact, an AI-based model of HealthMap, at Boston Children’s Hospital (USA), sounded one of the first alarms on 30 December 2019, around 30 minutes earlier than a scientist at the Program for Monitoring Emerging Diseases (PMED) issued an alert (see the discussion in Naudé 2020). For the further tracking and prediction of how COVID-19 will spread, however, AI has so far not been very useful. This is for a number of reasons. The first is that AI requires data on COVID-19 to train. An example of how this can be done is the case of the 2015 Zika- virus, whose spread was ex post predicted using a dynamic neural network (Akhtar et al. 2019). Because COVID-19 is different from Zika, or other infections, and because there are at the time of writing still not sufficient data to build AI models that can track and forecast its spread. Most of the growing number of publications reporting on using AI for diagnostic and predictive purposes so far tend to use small, possibly biased, and mostly Chinese-based samples, and have not been peer-reviewed.

A number of promising initiatives, however, have been started to gather and share data -both existing data, new data, and to train new AI models. These include the World Health Organization’s (WHO) Global Research on Coronavirus Disease Database, which also provides links to other similar initiatives. One of these is the open access data of the GISAID Initiative (formerly the Global Initiative on Sharing All Influenza Data). Amongst other initiatives, perhaps the most ambitious is the joint initiative between Semantic Scholar, the Allen Institute for Artificial Intelligence, Microsoft, Facebook, and others, to make openly available the COVID- 19 Open Research Dataset (CORD-19) which contains around 44,000 scholarly articles for data mining.

Kaggle, a data science competition platform, has issued a data competition based on this data, a COVID-19 Open Research Dataset Challenge. And contributing to the need for more (accessible) data, Elsevier made publicly available in its Novel Coronavirus Information Center early-stage and peer-reviewed research on COVID-19 and to around 20,000 related articles on ScienceDirect, as well as the full texts for data mining. Similarly, The Lens has made available all its data on patents in what it calls the Human Coronavirus Innovation Landscape Patent and Research Works Open Datasets to support the search for new and repurposed drugs. And Chen et al. (2020a) published the first public COVID-19 Twitter dataset.

There is a second reason why AI has so far not been very useful in tracking and predicting the spread of the disease. It is not only a lack of historical training data but also due to problems with using “big data”, e.g., such as harvested from social media. The pitfalls of big data and AI in the context of infectious diseases was illustrated in the infamous failure of Google Flu Trends. Lazer et al. (2014) referred to these as “big data hubris and algorithm dynamics”. For instance, as the infection continues to spread, the social media traffic around it accumulates, so the amount of noise accumulates which has to be filtered through before meaningful trends can be discerned. Generally, and this is also bad news for AI forecasting models in other fields, including economics and finance, since for any prediction algorithm that rely on past behavior, a global outlier event with its mass of new and unprecedented data, such as COVID-19, can be described as Rowan (2020) does as “the kryptonite of modern Artificial intelligence”. As a result, he concludes that over the near future “many industries are going to be pulling the humans back into the forecasting chair that had been taken from them by the models”.

Furthermore, scientists will need to deal with the deluge of scientific papers and new data being generated, and shift through these. More than 500 scientific articles on the pandemic now appear daily (see Gruenwald et al. 2020). This potential information overload is, however, where data analytic tools can play an important role. An example of an initiative in this regard is the *COVID*-*19 Evidence Navigator* by Gruenwald et al. (2020) which provides computer-generated evidence maps of scientific publications on the pandemic, daily updated from PubMed.

As a result of a lack of data, noisy social media and outlier data, big data hubris, and algorithmic dynamics, AI forecasts of the spread of COVID-19 are not yet very accurate or reliable (Naudé 2020). Hence, so far, most models used for tracking and forecasting do not use AI methods. Instead, most forecasters prefer established epidemiological models, so-called SIR models Song et al. (2020). For example, the Robert Koch Institute in Berlin uses an epidemiological SIR model that takes into account containment measures by governments, such as lockdowns, quarantines, and social distancing prescriptions. Its model has been applied to China to illustrate that containment can be successful in reducing the spread to slower than exponential rates—see Maier and Brockmann (2020).

To track COVID-19’s spread in real time, a veritable industry of data “dashboard” creation, for visualization of the disease, has emerged. The first, and most frequently used, is that of the John’s Hopkins’ Center for Systems Science and Engineering (CSSE) which is described in Dong et al. (2020). The data collected and made available through this dashboard is available on a GitHub repository, at https://github.com/CSSEGISandData/COVID-19.

*MIT Technology Review* has produced a ranking of these tracking and forecasting dashboards and to facilitate the production of data visualizations and dashboards of the pandemic, *Tableau* has created a *COVID*-*19 Data Hub with* a COVID-19 Starter Workbook. Sarkar (2020) provides a Python script to illustrate how one could extract data from the New York Times’ COVID-19 dataset and create data visualizations of the progression of the infection. The emergence of dozens of dashboards and visualizations of COVID-19 has however also led to calls for responsible visualization of COVID-19 data, see e.g. Makulec (2020).

## Diagnosis and prognosis

In addition to potentially tracking and predicting the spread of COVID-19, AI can also be used in the diagnosis and prognosis of the disease. In fact, this is perhaps where most of the first rush of AI initiatives focused on. Fast and accurate diagnosis of COVID-19 can save lives, limit the spread of the disease, and generate data on which to train AI models. There is growing effort to train AI models to diagnose COVID-19 using chest radiography images. According to a recent review of AI applications against COVID-19 by Bullock et al. (2020), argues that AI can be as accurate as humans, can save radiologists’ time, and perform a diagnosis faster and cheaper than with standard tests for COVID-19. Both X-rays and computed tomography (CT) scans can be used. Representative contributions in this regard include Chen et al. (2020b) and Wang and Wong (2020). The latter developed *COVID*-*Net*, a deep convolutional neural network (see e.g. Rawat and Wang (2017)), which can diagnose COVID-19 from chest radiography images. It has been trained on open repository data from around 13,000 patients with various lung conditions, including COVID-19. However, as the authors indicate, it is “by no means a production-ready solution”, and they call on the scientific community to develop it further, in particular to “improve sensitivity” (Rawat and Wang 2017, p. 6).

Given that not all people diagnosed with COVID-19 will need intensive care, the ability to be able to forecast who will be affected more severely can help in targeting assistance and planning medical resource allocation and utilization. Yan et al. (2020) used Machine Learning to develop a *prognostic prediction algorithm* to predict the mortality risk of a person that has been infected, using data from (only) 29 patients at Tongji Hospital in Wuhan, China. And Jiang et al. (2020) presents an AI that can predict with 80% accuracy which person affected with COVID-19 may go on to develop acute respiratory distress syndrome (ARDS). The sample that they used to train their AI system is, however, small (only 53 patients) and restricted to two Chinese hospitals.

Largely, the potential of AI is diagnosis is not yet carried over into practice, although it has been reported that a number of Chinese hospitals have deployed “AI-assisted” radiology technologies. Radiologists elsewhere have expressed their concern that there is not enough data available to train AI models, that most of the available COVID-19 images come from Chinese hospitals and may suffer from selection bias, and that using CT-scans and X-rays may contaminate equipment and spread the disease further. Indeed, the use of CT scans in European hospitals has dropped after the pandemic broke, perhaps reflecting this concern (Ross and Robbins 2020). It is probably correct as Coldeway (2020) concludes, “No one this spring is going to be given a coronavirus diagnosis by an AI doctor”. It also seems that comparatively less effort is on using AI for very early diagnostic purposes, for instance, in identifying whether someone is infected before it shows up in X-rays or CT scans, or on finding data-driven diagnostics that have less contamination risk.

## Treatments and vaccines

A third area where AI can potentially make a contribution in the fight against COVID-19 is in identifying possible treatments and vaccines. Even long before the COVID-19 outbreak, AI was lauded for its potential to contribute to new drug discovery, see e.g. Coldeway (2019), Fleming (2018), Segler et al. (2018) and Smith (2018). In the case of COVID-19, a number of research labs and data centers have already indicated that they are recruiting AI to search for treatments for and a vaccine against COVID-19. The hope is that AI can accelerate both the processes of discovering new drugs as well as for repurposing existing drugs. A number of researchers have already reported discovering drugs for repurposing. These include Beck et al. (2020) who report results from using Machine Learning to identify that an existing drug, *atazanavir*, could potentially be repurposed to treat COVID-19, and Stebbing et al. (2020), who identified *Baricitinib,* used to treat rheumatoid arthritis and myelofibrosis, as a potential treatment for COVID-19.

It is not very likely that these treatments (in particular a vaccine) will be available in the near future, at least to be of much use during the current pandemic. The reason is that the medical and scientific checks, trails, and controls that need to be performed before these drugs will be approved, once they have been identified and screened, will take time—according to estimates up to 18 months for a vaccine (Regalado 2020). See also Vanderslott et al. (2020) for an explanation of the process that a potential anti-COVID-19 drug will have to go through.

## Social control

A fourth role for AI in fighting the COVID-19 pandemic is in social control. AI has been argued to be necessary to manage the pandemic by using thermal imaging to scan public spaces for people potentially infected, and by enforcing social distancing and lockdown measures (Rivas 2020). For example, as described by Chun (2020) “At airports and train stations across China, infrared cameras are used to scan crowds for high temperatures. They are sometimes used with a facial recognition system, which can pinpoint the individual with a high temperature and whether he or she is wearing a surgical mask.” It is reported that these cameras can scan 200 persons per minute and will recognize those whose body temperature exceeds 37.3° (Dickson 2020). Thermal imaging has, however, been criticized as being inadequate to identify from a distance a fever in people who are wearing glasses (because scanning the inner tear duct gives the most reliable indication) and because it cannot identify whether a person’s temperature is raided because of COVID-19 or some other reason (Carroll 2020).

However, as Chun (2020) worryingly reports, “This system is also being used to ensure citizens obey self-quarantine orders. According to reports, individuals who flouted the order and left home would get a call from the authorities, presumably after being tracked by the facial recognition system”. This type usage is not limited to China. A USA computer vision-based startup is already offering “social distancing detection” software, which uses camera images to detect when social distancing norms are breached, after which it will send out a warning (Maslan 2020). At the time of writing, most advanced economies have been considering and/or testing various contact tracing apps and related tools to provide social control, see for instance the discussion in Gershgorn (2020). 

Whereas using AI to predict and diagnose COVID-19 is hampered due to lack of historical training data, AI tools such as computer vision and robots are not. Therefore, we are more likely over the short term to see this type of AI being used and used moreover for social control. Related technologies, such as mobile phones with AI-powered apps or wearables that harvest location, usage, and health data of their owners, are also more likely to be employed. According to Petropoulos (2020) such apps can “enable patients to receive real-time waiting-time information from their medical providers, to provide people with advice and updates about their medical condition without them having to visit a hospital in person, and to notify individuals of potential infection hotspots in real-time so those areas can be avoided”.

Useful as these are, the fear is that once the outbreak is over, that erosion of data privacy would not be rolled back and that governments would continue to use their improved ability to survey their populations- and use the data obtained in the fight against COVID-19 for other purposes. As Harari (2020) warns “Even when infections from coronavirus are down to zero, some data- hungry governments could argue they needed to keep the biometric surveillance systems in place because they fear a second wave of coronavirus, or because there is a new Ebola strain evolving in central Africa, or because…you get the idea”.

## Concluding remarks

In conclusion, AI has the potential to be a tool in the fight against COVID-19 and similar pandemics. However, from the above rapid scan of the current state of play, one has to concur with as Petropoulos (2020) that “AI systems are still at a preliminary stage, and it will take time before the results of such AI measures are visible”. Bullock et al. (2020) in one of the first surveys of AI models used against COVID-19 agrees, concluding that “very few of the reviewed [AI] systems have operational maturity at this stage.”

Clearly, data is central to whether AI will be an effective tool against future epidemics and pandemics. The fear is that public health concerns would trump data privacy concerns.  Mission creep may occur, with governments continuing the extraordinary surveillance of their citizens long after the pandemic is over. Thus, concerns about the erosion of data privacy are justified.

Given the public health threat posed by the pandemic, the European GDPR (Article 9) allows personal data collection and analysis, as long as it has a clear and specific public health aim (Ienca and Vayena 2020). Flexibility to gather and analyze big data promptly is essential in combatting the pandemic, even if it may require that the authorities collect more personal data than many people would feel comfortable with. Therefore, it is crucial that the authorities take particular care in their handling of such data and their justifications and communications to the public at large. The danger is that the people could lose trust in government, which will, as Ienca and Vayena (2020, p. 1) pointed out, “make people less likely to follow public-health advice or recommendations and more likely to have poorer health outcomes”.

Finally, although AI’s use has so far been rather limited, the pandemic and the policy responses to it may accelerate the digitalization of the economy, including the move towards greater automation of human labor, the re-shoring of production activities, and growing market dominance by a few large digital platform firms. As such, the innovations in AI technology that may be an outcome of the present crisis, may require of society to make faster progress to lay down appropriate mechanisms for the governance of AI.
